# Improve follicular thyroid carcinoma diagnosis using computer aided diagnosis system on ultrasound images

**DOI:** 10.3389/fonc.2022.939418

**Published:** 2022-11-16

**Authors:** Huan Zheng, Zebin Xiao, Siwei Luo, Suqing Wu, Chuxin Huang, Tingting Hong, Yan He, Yanhui Guo, Guoqing Du

**Affiliations:** ^1^ Department of Ultrasound, Guangdong Provincial People’s Hospital, Guangdong Academy of Medical Sciences, Guangzhou, China; ^2^ Department of Pathology, Guangdong Provincial People’s Hospital, Guangdong Academy of Medical Sciences, Guangzhou, China; ^3^ Department of Computer Science, University of Illinois Springfield, Springfield, IL, United States

**Keywords:** follicular thyroid carcinoma, computer aided diagnosis, deep learning, thyroid ultrasonography, transfer learning

## Abstract

**Objective:**

We aim to leverage deep learning to develop a computer aided diagnosis (CAD) system toward helping radiologists in the diagnosis of follicular thyroid carcinoma (FTC) on thyroid ultrasonography.

**Methods:**

A dataset of 1159 images, consisting of 351 images from 138 FTC patients and 808 images from 274 benign follicular-pattern nodule patients, was divided into a balanced and unbalanced dataset, and used to train and test the CAD system based on a transfer learning of a residual network. Six radiologists participated in the experiments to verify whether and how much the proposed CAD system helps to improve their performance.

**Results:**

On the balanced dataset, the CAD system achieved 0.892 of area under the ROC (AUC). The accuracy, recall, precision, and F1-score of the CAD method were 84.66%, 84.66%, 84.77%, 84.65%, while those of the junior and senior radiologists were 56.82%, 56.82%, 56.95%, 56.62% and 64.20%, 64.20%, 64.35%, 64.11% respectively. With the help of CAD, the metrics of the junior and senior radiologists improved to 62.81%, 62.81%, 62.85%, 62.79% and 73.86%, 73.86%, 74.00%, 73.83%. The results almost repeated on the unbalanced dataset. The results show the proposed CAD approach can not only achieve better performance than radiologists, but also significantly improve the radiologists’ diagnosis of FTC.

**Conclusions:**

The performances of the CAD system indicate it is a reliable reference for preoperative diagnosis of FTC, and might assist the development of a fast, accessible screening method for FTC.

## 1 Introduction

Follicular thyroid cancer (FTC) accounts for 11.5%-13.4% of differentiated thyroid cancer (DTC) patients (1, 2). FTC is more likely to develop distant metastasis and has a higher mortality rate than papillary thyroid cancer (PTC). Survival in FTC patients was 77.0% at 10 years, and 33.7% at 20 years (3). Although collectively referred to as DTC, if FTC and PTC are combined to study, the disease characteristics of FTC will be occluded by PTC. Therefore, it is necessary to study FTC alone.

Meanwhile, the diagnosis of FTC is more challenging than PTC and other types of thyroid cancer. For follicular patterned thyroid lesions, ultrasound is difficult to distinguish them among FTC, thyroid follicular adenoma (FA), and follicular adenomatoid (hyperplastic) nodules. The commonly used thyroid ultrasound image classification evaluation system (such as American College of Radiology Thyroid Imaging Reporting and Data System, short for ACR TIRADS) is not reliable for the identification of follicular tumors. Neither fine needle aspiration biopsy (FNA) can provide an accurate diagnosis of benign and malignant in follicular tumors. Even frozen sections are not suitable for the diagnosis of follicular tumors, because the detection rate of capsules or blood vessel infiltration in a single frozen section is very low in the case of FTC (4).

Nowadays, various computer aided diagnosis (CAD) systems using thyroid ultrasound (TUS) images present excellent performances in treating with PTC. For example, the classification accuracy of the CAD model for thyroid nodule proposed by Chi et al. reached 99.10%, the sensitivity 99.10%, and the specificity 93.90% (5). However, there are only two studies of CADs focusing on differentiating follicular neoplasm on TUS before, and their diagnostic performances of FTC are not as satisfying as that of PTC. Seo et al. conducted an image recognition model using a convolutional neural network (CNN) that concentrated on capturing the features of the boundary region of thyroid follicular neoplasms and disregarded features of intro area of thyroid nodule images, achieving the positive predictive value (PPV) of 67.49% (27/40), the sensitivity of 71.05% (27/38), and the accuracy of 89.52% (205/229). This study used only 39 FAs and 39 FTCs to train the CNN model, and, with these extremely small training data sets and their model, tested 191 FAs and 38 FTCs (6). Yang et al. took the whole lesions of follicular neoplasms into account in their CNN model, as a result, the classification accuracy of FTC and FA was improved to 96%. There were 830 images included in this study for training and validation, without the exact information of FA/FTC ratio (7).

Efforts on CAD systems to recognize FTC are far from enough and effective preoperative diagnosis of FTC is needed, because it could not only avoid excessive diagnostic surgical resection, but also avoid treating FTC as a benign tumor. Therefore, aiming to identify FTC on TUS, we have developed a novel CAD system based on deep learning on an ultrasound image for FTC diagnosis. Our study on CAD of FTC is different from previous CAD works. We not only simply propose a CAD system for FTC and justify its performance on different subsets of the TUS images, but also verifies its efficiency on facilitating and improving the performance of physicians with different levels of experience. The new contribution of this study includes two folders: 1) a novel CAD system for FTC diagnosis is proposed using a transfer learning on a deep residual convolutional neural network (CNN); 2) clinical experiments are studied to justify the performance improvement of radiologists with CAD’s help. In addition, we consider adenomatoid nodules besides FA and FTC, which is confusing to classify with follicular neoplasms on TUS and cytology, and we balanced the dataset of benign and malignant cases.

The rest of the paper is organized as: the proposed method is given in Section 2, Section 3 demonstrates the experimental results and comparative analysis of different methods, and a detailed discussion is given in Section 4. Finally, Section 5 concludes the whole paper.

## 2 Materials and methods

### 2.1 Materials

A total of 1159 images were obtained from 412 patients at Guangdong Provincial People’s Hospital from January 1st, 2012, to December 12th, 2020, consisting of 351 images from 138 FTC patients, and 808 images from 184 FA patients and 90 patients with adenomatoid nodules. All of the patients included have been performed TUS examination, thyroid resection and paraffin pathological diagnosis in hospital. All samples have been categorized into two classes: malignant class (FTC images), and benign class (FA images and adenomatoid nodule images). **Table 1** shows the basic characteristics of subjects studied. Among 412 patients, 274 are benign cases and 138 are in malignant group. In benign group, 89 are male and 185 are female, and they are 44.12 ± 13.26 years old and nodule size is averagely 3.88 ± 1.48 cm. Among the malignant cases, 31 are male and 107 are female, and their age is 43.85 ± 15.78 years old and the average nodule size is 3.74 ± 1.97 cm.

**Table 1:** Characteristics of study subjects.

	Benign group (n = 274)	Malignant group (n = 138)
Gender		
Male	89(32.48%)	31(22.46%)
Female	185(67.52%)	107(77.54%)
Age (y)	44.12 ± 13.26	43.85 ± 15.78
Nodule size (cm)	3.88 ± 1.48	3.74 ± 1.97

Ultrasound images acquisition: The US scans were performed by radiologists with 1–18 years of experience in thyroid imaging with a 7–12 or 6-13 or 7-14 MHz linear array probe of different ultrasound machines, including HITACHI Hi Version Ascendus, HITACHI Hi Version Preirus, HITACHI Hi Version Avius, TOSHIBA Aplio 500, TOSHIBA Aplio 400, Mindray Resona 8, Philips EPIQ 7, Supersonic Image Aixplorer^®^. Image quality requirements are as follows: 1. Gray scale ultrasound images are in JPG format and single-frame, and dual-frame images need to be cut in two; 2. Images mustn’t be covered with any manual mark, such as arrowhead, measurement line, annotation, etc. Specially, the US images needn’t any manual segmentation.

### 2.2 Proposed method

**Figure 1:**
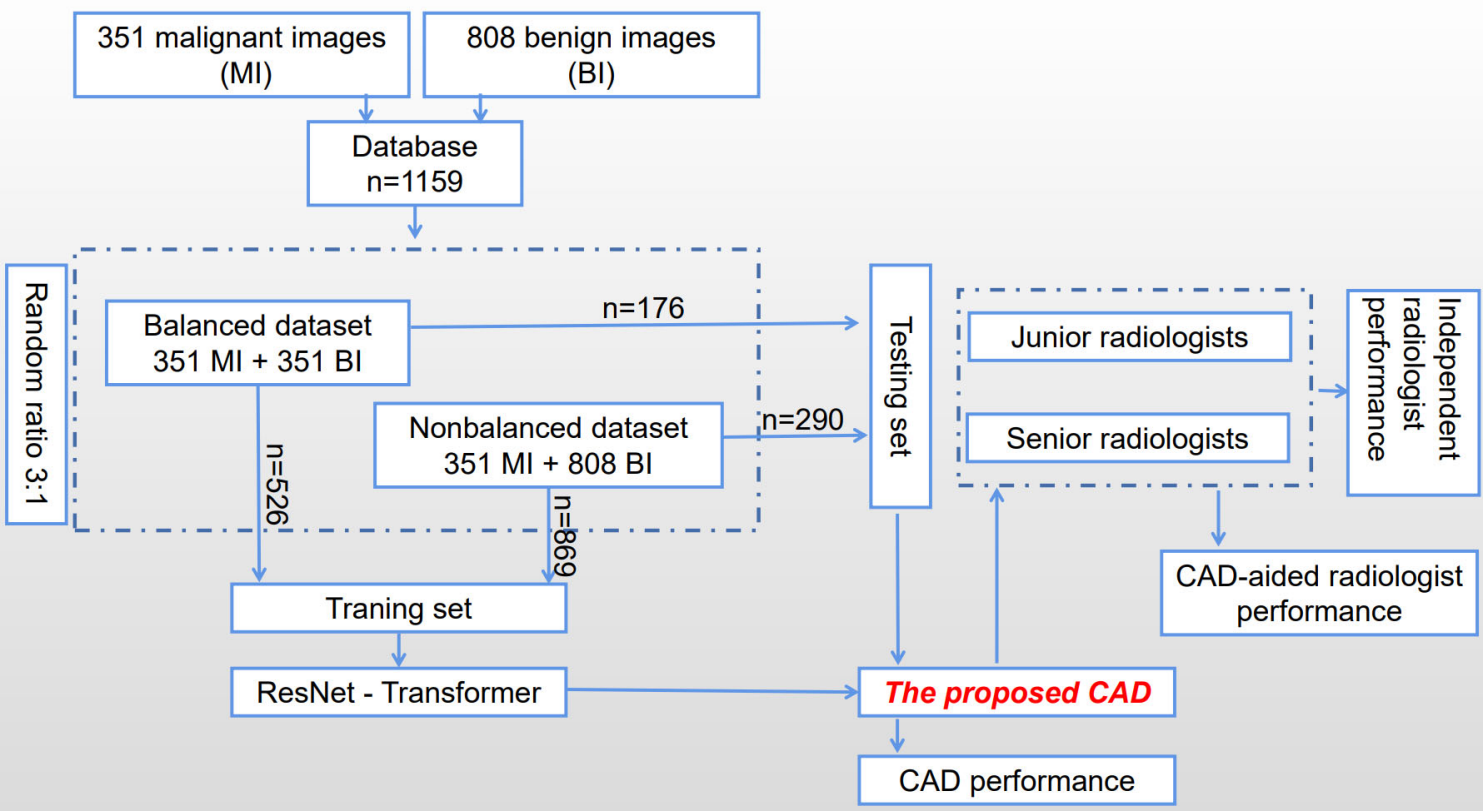
Flowchart of the construction and validation of the proposed CAD.

The process of constructing and validating the proposed CAD system is shown in **Figure 1** briefly. A database of 1159 images has been collected consisting of 351 malignant images (MI) and 808 benign images (BI), and then was divided into two subsets according to the ratio between benign and malignant groups to train and test the proposed CAD system, which is developed based on a transfer learning of a residual network (ResNet) to extract features on TUS. Six radiologists with different experiences participated in the experiments to verify whether and how much the proposed CAD system helps to improve their diagnostic performance.

In the proposed CAD system, a deep residual network is redesigned by changing the architecture and output parts to classify the TUS images. Firstly, basic layers in CNN are introduced as follows.

#### 2.2.1 CNN

In deep learning networks, multiple layers are stacked one by one, and the output of one layer becomes the input for the following layer. A convolution layer is a basic layer where different filters perform a convolution operation to extract the features from the input images or the feature maps of former layers with different kernels (8). The kernel weights are tuned using a gradient descent approach with backpropagation. Different activation functions such as rectified linear units (ReLU), Sigmoid, Tangent, and softmax functions, are used in activation layers to convert the nonlinear values into linear values. Max pooling, Average pooling, Global Max pooling, and Global Average pooling methods are used in the pooling layers to reduce the redundant parameters. The fully connected layer is usually directed to the final output layer where each neuron is connected from the previous layer. The outputs of the fully connected layers are interpreted as the estimated probability of the input images belonging to a certain class. A global average pooling layer calculates the spatial average of the feature maps from the previous layer as the confidence to different categories, which is more meaningful and interpretable because it corresponds to feature maps with categories.

A residual network (ResNet) (9) proposed skip connections, or short-cuts, to jump over some layers. Typically it consists of convolutional layers, rectified linear units (ReLU) layers, and layer skips.

#### 2.2.2 Proposed transfer learning network

Transfer learning approach redesigns the pre-defined network to make it finish different classification tasks, which is able to reduce the time in training and improves the network’s generalization ability. In our proposed transfer learning network, rather than building a model from scratch, a pre-trained ResNet50 network, which was trained using ImageNet, is selected as a backbone to extract the features from TUS images. Inside it, a fully connected layer is modified to match the output numbers of classified categories, and a binary cross-entropy function is used as the loss function which computes the binary cross-entropy (BCE) between predictions and targets (10). The original ResNet is improved by adding a fully connected layer for feature extraction and adding a global average pooling to interpret these features in the classification task. The idea is to generate one feature map for each corresponding category of the classification task in the last convolutional layer. Thus the feature maps can be interpreted as categories confidence maps. Also, the global average pooling is a structural regularizer to prevent overfitting for the overall structure.

#### 2.2.3 Gradient-weighted class activation mapping

The proposed CAD system aims to help radiologists by providing both the classification results and the salient features on TUS images. The gradient-weighted class activation mapping (Grad-CAM) method generates an activation map that highlights the crucial areas (11). In the Grad-CAM method, the gradients of the layers after the final convolutional layer produce a map to highlight important areas, where each neuron is assigned significance values which respondents to class-specific information. The proposed CAD system uses the Grad-CAM method to provide the visuality by focusing attention on the critical lesion regions on TUS images.

## 3 Results

### 3.1 Platform settings

The modified deep learning model was trained on a server with a 2 x Six-Core Intel Xeon processor and 128GB of memory. The server is equipped with an NVIDIA Tesla K40 GPU with 12GB of memory.

**Figure 2:**
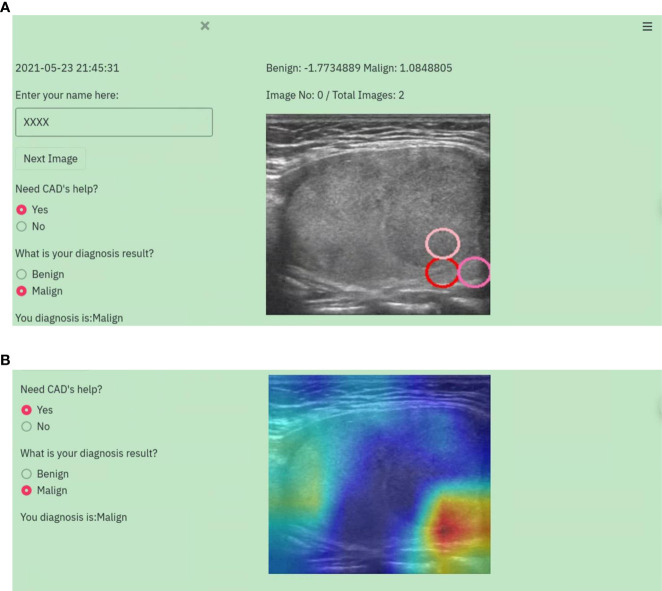
An example of the diagnosis procedure with the WGUI. **(A)** Diagnosis interface with the diagnosis results from the CAD. **(B)** Diagnosis interface with Gad-CAM map.

To verify the efficiency of the proposed CAD system, a web-based graphic user interface (WGUI) program was designed to provide a platform for radiologists to perform diagnoses on TUS images using the help of CAD. This WGUI randomly selects images from the dataset, displays them in front of radiologists, and provides diagnosis options for radiologists to select. It also assists radiologists by providing the diagnosis results of CAD, displaying color Grad-CAM maps, and marking salient feature reference regions. An example is shown in **Figure 2** in demonstrating the diagnosis procedure with the WGUI.

### 3.2 Image dataset settings

In the TUS dataset, to evaluate the classification performance on the datasets with different ratios of two categories, images have been selected twice to construct a balanced dataset and an unbalanced dataset. There are 351 malignant images, 351 benign images and 702 images in total in the balanced dataset, with each category represented by the same number of images. The unbalanced dataset included all images without considering the balanced among different categories, in which there are 351 malignant images, 808 benign images and 1159 images as a whole.

### 3.3 Evaluation metrics

A confusion matrix is used to evaluate classification performance. In it, each row represents the instances of a predicted class, and each column represents the instances of an actual class. Given that a row of the confusion matrix corresponds to a specific true value, two evaluation metrics, precision, and recall (sensitivity) are calculated for multiple classifications as follows:


(1) $$\text{Precision}\left(\text{i}\right)=\frac{M_{ii}}{\sum_{j}M_{ji}}$$

(2) $$\text{Recall}\left(\text{i}\right)=\frac{M_{ii}}{\sum_{j}M_{ij}}$$

where Precision(i) is the fraction of samples where the algorithm correctly predicted class *i* out of all predictions using the algorithm, and Recall(i) is the fraction of cases where the algorithm correctly predicted i out of all the true cases of *i*. *M_ij_* the samples whose true class is *i* and prediction class is *j*.

F1-score is also used to evaluate classification performance and defined as:


(3) $$\text{F}1\left(\text{i}\right)=2\times \frac{\text{Precision}\left(\text{i}\right)\times \text{Recall}\left(\text{i}\right)}{\text{Precision}\left(\text{i}\right)+\text{Recall}\left(\text{i}\right)}$$

Accuracy is one metric for evaluating classification performance, which is defined as a fraction of correct predictions out of total predictions as:


(4) $$\text{Accuracy}=\frac{Number\;of\;correct\;predictions}{Total\;number\;of\;predictions}=\frac{\sum_{i}M_{ii}}{\sum_{ij}M_{ij}}$$

### 3.4 Evaluation results on the balanced dataset

In the balanced dataset where each category was represented by the same number of images, 702 images were selected randomly from the whole dataset. In the balanced dataset, 75% of the images were used for training and 25% for testing. 526 images were in the training set, and 176 in the testing set.

**Figure 3:**
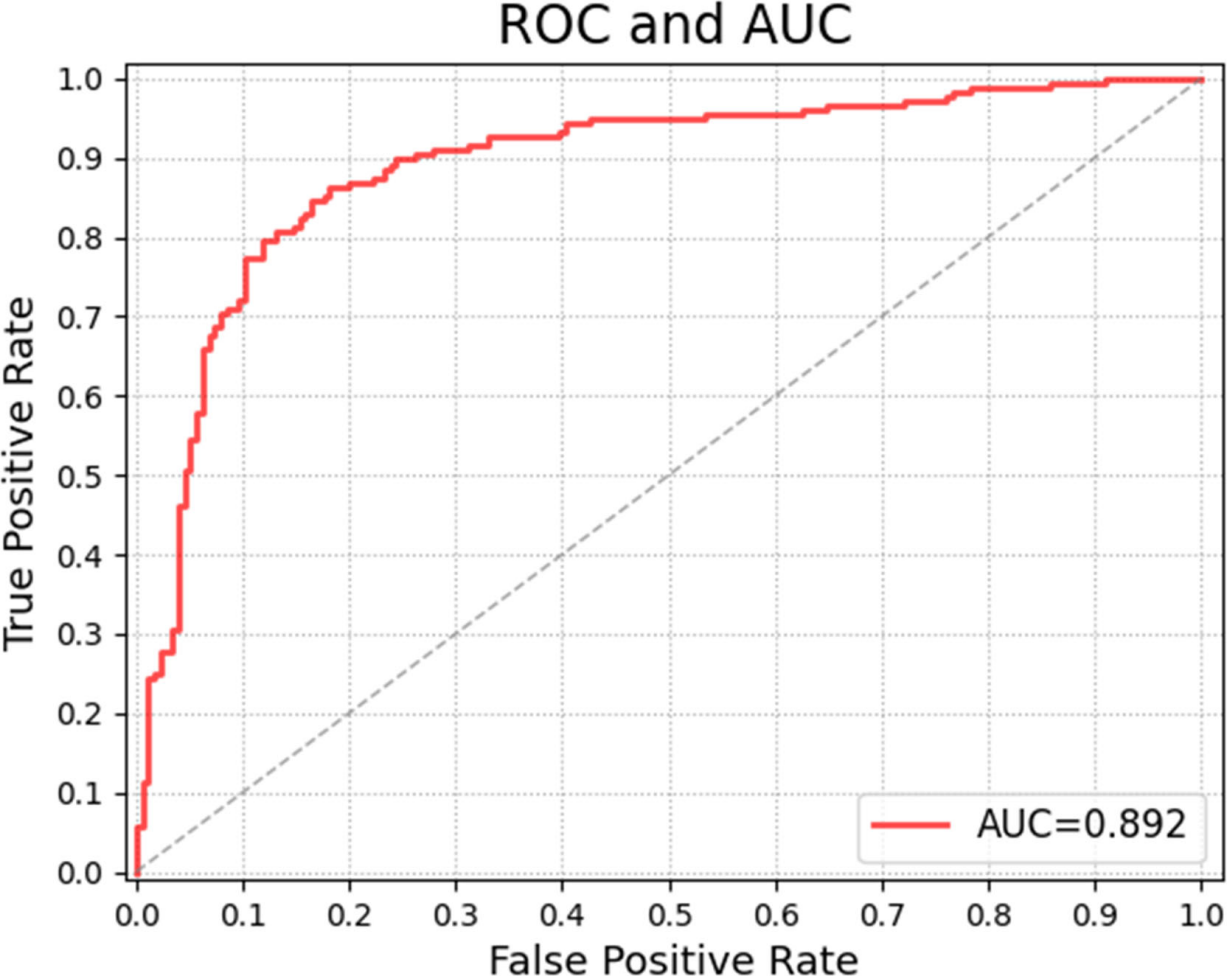
ROC curve of the proposed model on balanced data set.

**Figure 4:**
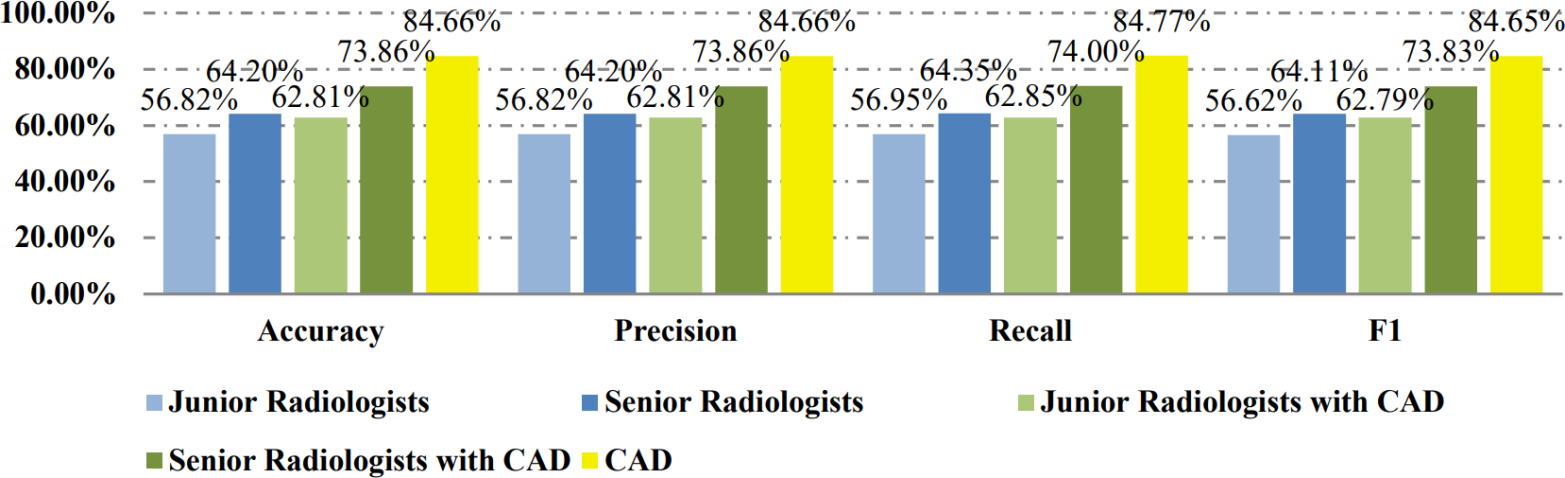
Evaluation results for CAD and different radiologists on the balanced data set.

**Table 2** shows the confusion matrices on the proposed CAD approach. In the tables of the confusion matrix, the rows correspond to the predicted class and the columns correspond to the true class. The diagonal elements correspond to correctly classified observations, and the off-diagonal cells correspond to incorrectly classified observations. Using the value from confusion matrix, Recall, Precision, and F1-score were calculated and shown in **Table 3**. **Figure 3** shows the receiver characteristics curve (ROC) and the area under ROC (AUROC) is 0.892. The proposed CAD system achieves precision, recall, and F1-score values of 87.50%, 82.80% and 85.08% for diagnosing benign cases, 81.82%, 86.75%, and 84.21% for malignant cases. These results of the evaluation metrics also indicate the proposed model achieves better performance than all radiologists shown in **Figure 4**.

**Table 2:** Confusion matrix of the proposed CAD on the balanced data set.

	Actual		
Predicted	Benign	Malign	Total
Benign	77	16	93
Malign	11	72	83
Total	88	88	

**Table 3:** Evaluation results of CAD on the balanced set.

Categories	Precision	Recall	F1-score
Benign	87.50%	82.80%	85.08%
Malign	81.82%	86.75%	84.21%
Average	84.66%	84.77%	84.65%
Total accuracy	84.66%		

**Table 4** shows the confusion matrices on the evaluations of the radiologists’ performance without and with the help of the proposed CAD. We employed two groups of radiologists with different levels of experience. Among them, three junior radiologists had less than 3 years’ experience and three senior radiologists had more than 5 years’ experience in TUS diagnosis. The values of Recall, Precision, and F1-score are compared in **Table 5** which are results of the radiologists with and without CAD’s helps. **Figure 4** shows evaluation results for the proposed CAD and different radiologists’ performance on balanced dataset.

**Table 4:** Confusion matrices between radiologists of different level on the balanced data set.

	Actual		
Predicted	Benign	Malign	Total
Junior radiologists			
Benign	168	132	300
Malign	96	132	228
Total	264	264	
Junior radiologists with CAD			
Benign	159	91	250
Malign	105	172	277
Total	264	263	
Senior radiologists			
Benign	183	108	291
Malign	81	156	237
Total	264	264	
Senior radiologists with CAD			
Benign	205	79	284
Malign	59	185	244
Total	264	264		

all numbers count all cases diagnosed by three radiologists.

**Table 5:** Evaluation results of radiologists on the balanced set.

	Junior Radiologists			Senior Radiologists			Junior Radiologists with CAD			Senior Radiologists with CAD		
Categories	Precision	Recall	F1-score	Precision	Recall	F1 score	Precision	Recall	F1 score	Precision	Recall	F1 score
Benign	63.64%	56.00%	59.57%	69.32%	62.89%	65.95%	60.23%	63.60%	61.87%	77.65%	72.18%	74.82%
Malign	50.00%	57.89%	53.66%	59.09%	65.82%	62.28%	65.40%	62.09%	63.70%	70.08%	75.82%	72.83%
Average	56.82%	56.95%	56.62%	64.20%	64.35%	64.11%	62.81%	62.85%	62.79%	73.86%	74.00%	73.83%
Total accuracy	56.82%			64.20%			62.81%			73.86%		

### 3.5 Evaluation with the unbalanced dataset

In the unbalanced dataset, all 1159 images were selected from the original dataset. The same ratio (75% and 25%) was used to split the dataset into the training and testing sets. From the 1159 images, 869 were selected for training and 290 for testing.

**Figure 5:**
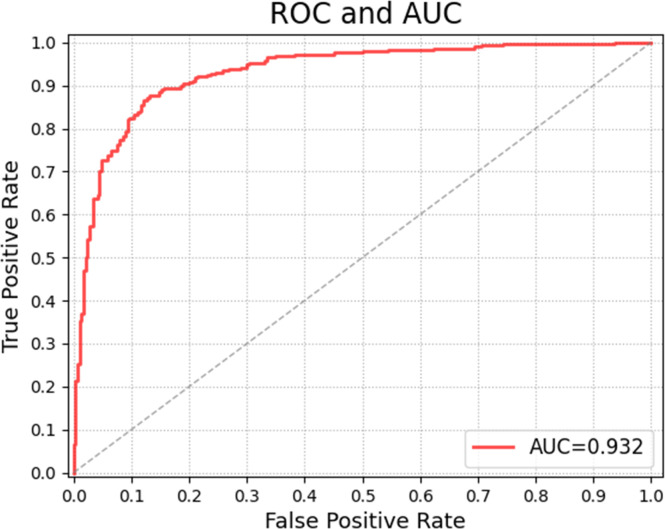
ROC curve of the proposed model on unbalanced data set.

**Figure 6:**
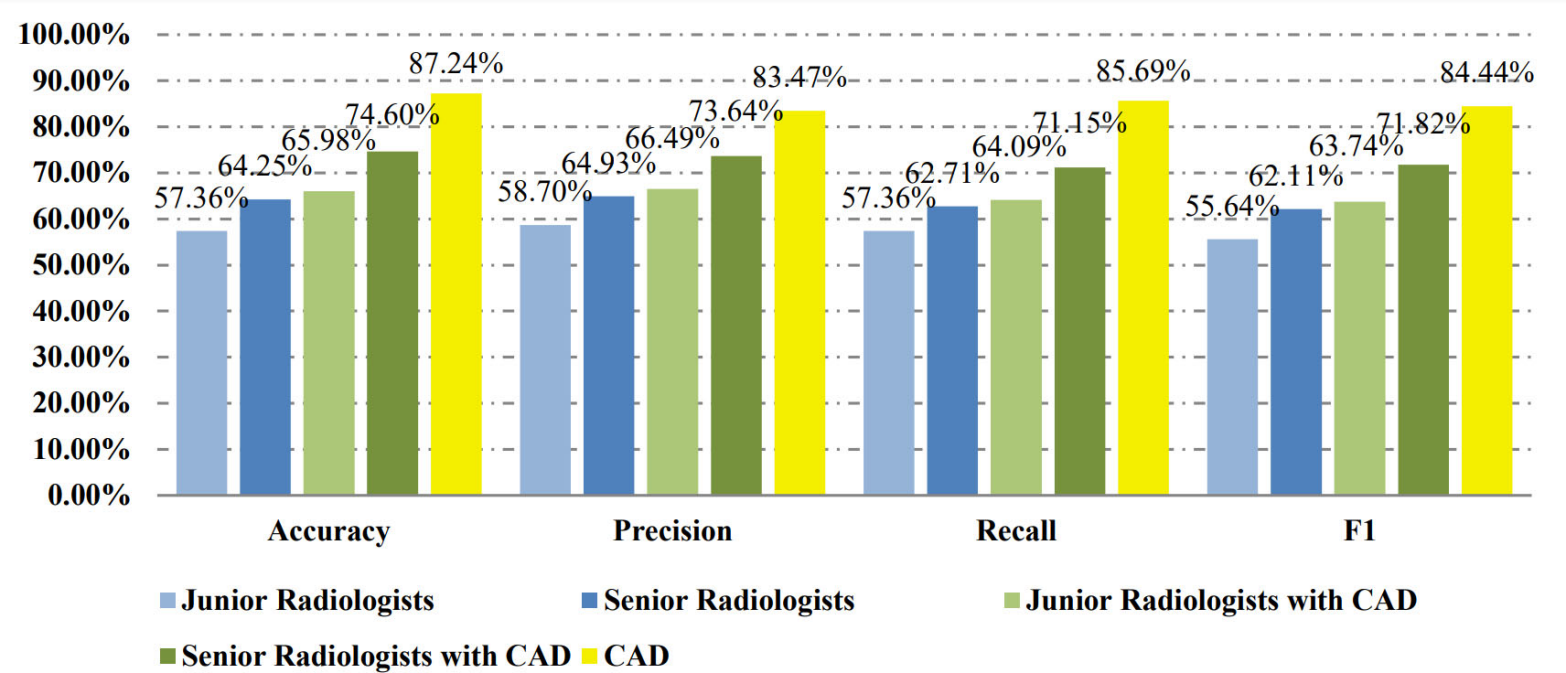
Evaluation results for CAD and different radiologists on the unbalanced data set.

**Tables 6** and **7** show the confusion matrices and results of the evaluation matrix of our proposed model on the unbalanced dataset. **Figure 5** shows the ROC and the value of AUROC is 0.932. **Table 8** shows the confusion matrices of different radiologists’ performance without and with the help of the proposed CAD on unbalanced dataset and **Table 9** shows their evaluation results. **Figure 6** compares valuation results for the proposed CAD and different radiologists’ performance on unbalanced dataset.

**Table 6:** Confusion matrix of the proposed model on the unbalanced data set.

	Actual		
Predicted	Benign	Malign	Total
Benign	188	23	211
Malign	14	65	79
Total	202	88	

**Table 7:** Evaluation results of CAD on the unbalanced set.

Categories	Precision	Recall	F1-score
Benign	93.07%	89.10%	91.04%
Malign	73.86%	82.28%	77.84%
Average	83.47%	85.69%	84.44%
Total accuracy	87.24%		

**Table 8:** Confusion matrices between radiologists of different level on the unbalanced data set.

	Actual		
Predicted	Benign	Malign	Total
Junior radiologists			
Benign	335	100	435
Malign	271	164	435
Total	606	264	
Junior radiologists with CAD			
Benign	395	85	480
Malign	211	179	390
Total	606	264	
Senior radiologists			
Benign	383	88	471
Malign	223	176	399
Total	606	264	
Senior radiologists with CAD			
Benign	461	76	537
Malign	145	188	333
Total	606	264	

all numbers count all cases diagnosed by three radiologists.

**Table 9:** Evaluation results on the unbalanced set.

	Three Junior Radiologists			Three Senior Radiologists			Three Junior Radiologists with CAD			Three Senior Radiologists with CAD		
Categories	Precision	Recall	F1-score	Precision	Recall	F1 score	Precision	Recall	F1 score	Precision	Recall	F1 score
Benign	55.28%	77.01%	64.36%	63.20%	81.32%	71.12%	65.18%	82.29%	72.74%	76.07%	85.85%	80.66%
Malign	62.12%	37.70%	46.92%	66.67%	44.11%	53.09%	67.80%	45.90%	54.74%	71.21%	56.46%	62.98%
Average	58.70%	57.36%	55.64%	64.93%	62.71%	62.11%	66.49%	64.09%	63.74%	73.64%	71.15%	71.82%
Total accuracy	57.36%			64.25%			65.98%			74.60%		

## 4 Discussion

The proposed CAD system using the transfer learning of the ResNet model was employed to identify FTC on TUS images. To evaluate the classification performance of the CAD and the ability to assist radiologists, a TUS dataset was collected, and six radiologists were involved in the verification experiments. Different images were selected to construct a balanced set and an unbalanced set to evaluate the classification performance on the dataset with different ratios of two categories. The proposed CAD system was trained and tested in the two datasets, respectively. The results demonstrate that the proposed CAD system accomplished high classification precisions on two sets. The quantitative metrics results also justify its better performance. The experimental results on the ability to assist radiologists demonstrated that the CAD system can significantly increase the radiologists’ performance on the FTC diagnosis on TUS.

Six radiologists participated in this study and were divided into junior and senior groups according to their experience on thyroid diseases diagnosis. Radiologists inside each group have similar prior experience levels, which can prevent bias due to experience inequalities. The performance in the identification of FTC lesions of senior radiologists was better than junior ones; however, the accuracy of both groups was not high. With the assistance of the proposed CAD system, junior radiologists achieved an accuracy similar to seniors, and both accuracies are improved significantly. This finding provides evidence of the CAD’s ability to help more physicians efficiently utilize TUS for FTC evaluation and follow-up. This finding has much practical significance.

Ultrasonography is limited in suggesting benign or malignant in follicular tumors, although previous studies have reached some positive conclusions. For example, Li et al. believed that an interrupted halo and satellite nodule(s) with or without halo ring are risk factors for FTC on ultrasound images (12), and Lee et al. found that US characteristics of rim calcifications help to differentiate FTC from FA (13). Those findings have limits to rely on, partly because FTC and FA are highly similar in histopathology, immunohistochemistry, genomics, and proteomics (14, 15), making the US features of the two sharing a large overlap. As a result, in human’s observation on TUS, FTC appears in similar sonographic characteristics to the benign category (16).

FNA is challenging to distinguish between FA, FTC and adenomatoid nodules, either. Combining TUS with FNA does not work well in this situation, leaving a lot of controversies in the clinical management of follicular pattern nodules (17). According to the promising results in our study, CAD may shed light on the diagnosis of FTC on TUS.

Several previous studies have demonstrated that AI classifier matches or exceeds radiologists while qualitatively analyzing thyroid nodules (18–20). It can easily identify and model a complicated nonlinear relationship in the image, and extract and quantify key image features, whereby image diagnosis converts from a subjective qualitative task to objective quantitative analysis and this might sharply reduce the differences in judgments among US professionals (21).

In our experiment, it was interesting that the performance of radiologists with the assistance of the CAD system on the diagnosis of FTC was still inferior to the proposed CAD system, which was contrary to the experiment designer’s assumption. It might be because the characteristics of many FTC cases are ambiguous which make the diagnosis subjective, and senior radiologists depend on more their experiences than the results by CAD, which are more objective and consistent, displaying remarkable advantages in diagnosis. Many factors hold the practice of AI models back in the real world, such as lack of explanation for conclusions from a black-box algorithm to solidify the trust among CAD systems, physicians and patients (22, 23). Humans indeed are required for oversight of AI’s algorithmic interpretation of images and data, because of its potential pitfalls and inherent biases which may increase systemic risks of harm, raise the possibility of errors with high consequences, and amplify complex ethical and societal issues (24).

It is insufficient for clinical decision-making with the computer alone. Humans are the main subject of medical practice. Some explanation techniques were used in our proposed CAD system to facilitate the utilization. The output of our CAD system was presented as diagnosing results with probability, highlighted critical lesion regions, and Grad-CAM maps. Clinicians can enter the field easily and give their verdicts according to the visualized diagnostic results by the CAD system as well as their own experience.

Currently, the diagnosis of FTC relies on a fairly complete pathological evaluation of the tumor envelope. It has great clinic significance accurately distinguish FTC from FA and adenomatoid nodules before surgery to avoid rashly performing thermal ablation. Further efforts will be taken, such as grading the risk of suspicious follicular tumors based on image characteristics and providing relevant suggestions to distinguish what kind of nodules are not suitable for thermal ablation. CAD has a lot of room for development in these efforts.

Compared to the previous CNN models on FTC TUS, the total accuracy of our CAD system (84.66% on the balanced set and 87.24% on the unbalanced set) seems not outstanding, as Seo’s model achieved an accuracy of 89.52%, and Yang’s model gained an accuracy of 96%. However, the number of FAs is more than 5 times of FTCs in Seo’s testing set, and Yang’s testing set had no exact information about FA/FTC ratio (6, 7). The huger the number difference between the two compared groups is, the higher the accuracy is. Our testing sets are more balanced with FA/FTC 351/351 in the balanced set and 808/351 in the unbalanced set. What’s more, we consider adenomatoid nodules besides FA to differentiate with FTC, which is more confusing on TUS.

More improvements will be considered in future research. For this study is a single study in one hospital, there may be some differences on image acquirement in other hospitals and the model needs to be refined on different images, and prospective validation studies are also needed.

## 5 Conclusion

The proposed CAD system has promising performance in the diagnosis of FTC and is able to facilitate the diagnosis performance of radiologists with different levels. It is a reliable complement for TIRADS and FNA in preoperative diagnosis of follicular pattern nodules, which is challenging in clinic medicine. It also might assist the development of a fast, accessible screening method for thyroid diseases.

## Data availability statement

The raw data supporting the conclusions of this article will be made available by the authors, without undue reservation.

## Author contributions

Conceptualization: YG, GD, and HZ; investigation and data curation: HZ and ZX; methodology and formal analysis: YG and GD; validation: HZ, SL, SW, CH, TH, and YH; writing and editing: YG, GD, and HZ; resources and supervision: YG and GD. All authors contributed to the article and approved the submitted version.

## Conflict of interest

The authors declare that the research was conducted in the absence of any commercial or financial relationships that could be construed as a potential conflict of interest.

## Publisher’s note

All claims expressed in this article are solely those of the authors and do not necessarily represent those of their affiliated organizations, or those of the publisher, the editors and the reviewers. Any product that may be evaluated in this article, or claim that may be made by its manufacturer, is not guaranteed or endorsed by the publisher.
